# Detection of NASBA amplified bacterial tmRNA molecules on SLICSel designed microarray probes

**DOI:** 10.1186/1472-6750-11-17

**Published:** 2011-02-28

**Authors:** Ott Scheler, Lauris Kaplinski, Barry Glynn, Priit Palta, Sven Parkel, Kadri Toome, Majella Maher, Thomas Barry, Maido Remm, Ants Kurg

**Affiliations:** 1 Dept. of Biotechnology, Institute of Molecular and Cell Biology, University of Tartu, Tartu, Estonia; 2 Estonian Biocentre, Tartu, Estonia; 3 Dept. of Bioinformatics, Institute of Molecular and Cell Biology, University of Tartu, Tartu, Estonia; 4 Molecular Diagnostics Research Group, National Centre for Biomedical Engineering Science, National University of Ireland, Galway, Ireland

## Abstract

**Background:**

We present a comprehensive technological solution for bacterial diagnostics using tmRNA as a marker molecule. A robust probe design algorithm for microbial detection microarray is implemented. The probes were evaluated for specificity and, combined with NASBA (Nucleic Acid Sequence Based Amplification) amplification, for sensitivity.

**Results:**

We developed a new web-based program SLICSel for the design of hybridization probes, based on nearest-neighbor thermodynamic modeling. A SLICSel minimum binding energy difference criterion of 4 kcal/mol was sufficient to design of *Streptococcus pneumoniae *tmRNA specific microarray probes. With lower binding energy difference criteria, additional hybridization specificity tests on the microarray were needed to eliminate non-specific probes. Using SLICSel designed microarray probes and NASBA we were able to detect *S. pneumoniae *tmRNA from a series of total RNA dilutions equivalent to the RNA content of 0.1-10 CFU.

**Conclusions:**

The described technological solution and both its separate components SLICSel and NASBA-microarray technology independently are applicative for many different areas of microbial diagnostics.

## Background

The *ssrA *gene which encodes the tmRNA molecule has been identified in all known bacterial phyla [1,2]. The term tmRNA describes the dual "transfer" and "messenger" properties of this RNA molecule. In bacteria, the function of the tmRNA molecules is to release ribosomes that have become stalled during protein synthesis and to tag incomplete and unnecessary peptides for proteolysis. A typical tmRNA is between 300-400 nucleotides in size and is present in cells in relatively high copy number around 1000 copies per cell [3]. tmRNA molecules contain both conserved as well as variable regions between different species; complementary 3' and 5' ends fold together into a tRNA like structure that permits the entry to the ribosome when needed. Proteolysis-coding mRNA part and structural domains usually make up for the rest of the molecule. All those characteristics make the tmRNA transcript (and its *ssrA *gene) a suitable tool as a target marker molecule for phylogenetical analysis and species identification in microbial diagnostics. Over the last 10 years tmRNA and its corresponding gene have been used for species identification in several methods including fluorescence in situ hybridization (FISH) detection of specific bacteria [4], real-time PCR [5] and real-time NASBA [6] analysis of food and dairy contaminants and pathogen detection using biosensors [7]. Combining the capabilities of tmRNA for species identification with DNA microarray technology offers the potential to investigate samples simultaneously for large numbers of different target tmRNA molecules. DNA microarrays have found several practical applications in microbial diagnostics such as composition analysis and species identification of different environmental and medical samples as well as in microbial diversity investigation [8-10]. Depending on the experiment setup and specific probe design, precise detection of one specific microbe [11] or more complex analysis of microbial taxa can be performed [12]. The design of microarray probes for the detection of bacterial RNA poses unique challenges, because certain RNA/DNA or RNA/RNA mismatches have almost as strong binding affinity as matches [13]. The nearest-neighbor thermodynamic modeling (NN) approach should therefore be used to calculate the hybridization affinities (ΔG) of probes [14-16]. The hybridization on microarray surface is more complex then hybridization in solution and the NN model should include surface and positional parameters for more accurate modeling [17,18]. Although there are many recent studies of surface hybridization thermodynamics [19], the exact hybridization properties of microarray probes cannot be precisely modelled and experimental verification is still needed [20,21]. A common feature of many microarray analysis protocols is that the nucleic acid sequences of interest are amplified and labeled prior to the hybridization experiment. Hybridization protocols may involve labeled cDNA [22], cRNA [23] or (RT-)PCR products [24]. RNA molecules can also be amplified by Nucleic Acid Sequence Based Amplification (NASBA) [25]. Although not as common as RT-PCR, NASBA is less prone to genomic DNA contamination and therefore more suitable for applications where the testing of microbial viability is important [26]. Several methods have recently been published that describe different NASBA product labeling methods for the purpose of microarray hybridization. These methods include the dendrimer-based system NAIMA [27], biotin-streptavidin binding assisted labeling [28] and aminoreactive dye coupling to aminoallyl-UTP (aa-UTP) molecules in NASBA products [29]. In this report we present a complete technological solution for detection of low amounts of bacterial tmRNA molecules. We describe a new software program, SLICSel, for designing specific oligonucleotide probes for microbial diagnostics using nearest-neighbor thermodynamic modeling and evaluate SLICSel by testing the specificity of the designed tmRNA specific probes. Finally we demonstrate the sensitivity of these probes using a molecular diagnostics method that combines tmRNA amplification by NASBA with microarray-based detection [29]. Using this approach we were able to specifically detect *S.pneumoniae *tmRNA in the amount that corresponds to a single bacterium or less in the presence of 4000-fold excess of other bacterial tmRNA.

## Methods

### SLICSel program for probe design

The nearest-neighbor thermodynamic (NN) modeling of probe hybridization strength with target (specific hybridization) and control (nonspecific hybridization) nucleotide sequences at exact annealing temperature is used as design criterion of the SLICSel program. The previously published empirical formula was used to adjust the calculated thermodynamic values to the actual annealing temperature and salt concentration [15]. No surface and positional effects were added to the model to keep it universal and not bound to specific technology. We also expect that NN parameters on surface, although slightly different, are in correlation with the ones in solution [19].

### Bacterial strains

*Streptococcus pneumoniae *ATCC 33400 (*S.pneumoniae*), *Streptococcus pyogenes *ATCC 12344 (*S.pyogenes)*, *Klebsiella pneumoniae *ATCC 13883 (*K.pneumoniae*), *Moraxella catarrhalis *ATCC 25238 (*M.catarrhalis*) were obtained from DSMZ (Braunschweig, Germany); *Streptococcus agalactiae *(*S.agalactiae*) and Group C/G streptococcus (GrC/G) from University College Hospital (Galway, Ireland). Bacterial strains were grown in Brain Heart Infusion Broth (Oxoid, Hampshire, UK). Total RNA extraction and CFU counting is further described in the Additional file 1.

### Microarray design

We used the *S.pneumoniae *tmRNA molecule as the main specific target molecule, while tmRNAs from other bacteria were used as non-specific controls. The custom made microarray for SLICSel validation experiments contained 97 probes covering the whole *S.pneumoniae *tmRNA sequence. For NASBA-microarray experiment, the 25 best performing probes were selected and additional control probes specific to *S.pyogenes*, *S.agalactiae*, *M.catarrhalis *and *K.pneumoniae *(three for each) were also added. The precise probe list and microarray manufacturing have been described previously [30] and customization for the current article is described in the Additional file 1.

### *In vitro *tmRNA synthesis for validation experiment

For *in vitro *transcription of tmRNA *ssrA *genes of *S.pneumoniae*, *S.agalactiae*, *S.pyogenes*, Group C/G streptococcus, *M.catarrhalis *and *K.pneumoniae *were inserted in the pCR^® ^II-TOPO vector (Invitrogen, Carlsbad, CA, USA) under the transcriptional control of either T7 or SP6 promoter sequence. tmRNA molecules were transcribed from vector as described previously [30] with minor alterations. The complete protocol is available in the Additional file 1.

### NASBA amplification experiment

A series of experiments were performed to determine the detection capability of NASBA in combination with microarray hybridization. A NASBA primer pair (see the Additional file 1) was designed to amplify a 307 nucleotide tmRNA product using *S.pneumoniae *total RNA as a template. The T7 promoter was added to the forward primer in order to generate a sense strand of the RNA molecule. Three different amounts of *S. pneumoniae *total RNA were added to the NASBA reactions: 1 pg, 100 fg and 10 fg, corresponding to 10, 1 and 0.1 CFU, respectively. An equal volume of NASBA water (included in EasyQ kit) was added to control experiment without any *S. pneumoniae *total RNA. NASBA reactions were performed with NucliSENS EasyQ Basic kit v2 (bioMerieux bv, Boxtel, NL) according to manufacturer's instructions but with addition of aminoallyl-UTP (aa-UTP) as described previously [29]. Final concentration of aa-UTP (Epicentre, Madison, WI, USA) used in the reaction was 1 mM. EasyQ kit was used for 96 NASBA amplifications instead of the original 48 by halving all of the manufacturer suggested reagent volumes. In experiments with background RNA 10 pg of *S.pyogenes, S.agalactiae, M.cattarhalis *and *K.pneumoniae *total RNA were added, making the RNA excess ratios of each control to target RNA 10:1, 100:1 and 1000:1, respectively. Following amplification, tmRNA was purified using a NucleoSpin^® ^RNA CleanUp Kit and vacuum dried using RVC 2-25 CD rotational vacuum concentrator (Martin Christ GmbH, Osterode am Harz, Germany).

### Labeling of aa-UTP modified RNA and microarray hybridization

Extra amine groups of aa-UTP modified tmRNA molecules were labeled with the monoreactive fluorescent dye Cyanine™ 3-NHS (Cy3) (Enzo, Farmingdale, NY, USA) as described previously [30]. For the SLICSel validation experiments, 300 ng of *in vitro *synthesized target or control RNA was hybridized onto microarray. In NASBA experiments all of the amplified material was used in the subsequent microarray hybridization. In both cases vacuum dried RNA was resuspended in 80 μl of hybridization buffer and hybridized for 4 hours on the microarray in an automated HS-400 hybridization station (Tecan Austria, Grödig, Austria) at 55 C°. Complete hybridization protocol and reagents are shown in the Additional file 1. After hybridization, the slides were scanned using an Affymetrix 428 scanner (Affymetrix, Santa Clara, CA, USA), λ = 532 nm. Raw signal intensity data was analyzed using Genorama™ BaseCaller software (Asper Biotech, Estonia).

## Results

### Probe design software

SLICSel was used to design hybridization probes for all bacterial species in the experiment. It uses a brute-force algorithm that finds all theoretically acceptable probe sequences. All designed probes are guaranteed to have at least specified minimum difference (ΔΔG_control_) between the binding energies (ΔG) of specific and nonspecific hybridization and at most specified maximum binding energy difference (ΔΔG_target_) between the binding energies of the hybridization with different target sequences. The algorithm also accepts degenerate nucleotides in sequences; in which situation the worst-case variant is used (strongest binding for control set and weakest binding for target set). The program uses well-established thermodynamic models of hybridization in solution, as the more complex surface effects are still under active study and are also dependant on the microarray technology used. The program code can be easily extended to take account of more specific models, if needed. The tables for both DNA-DNA and DNA-RNA nearest-neighbor hybridization thermodynamics are included with the program. It is also possible to use a custom table of thermodynamic parameters, necessary if very specific experimental conditions are used. SLICSel is available from web interface at http://bioinfo.ut.ee/slicsel/

### SLICSel validation

**Figure 1:**
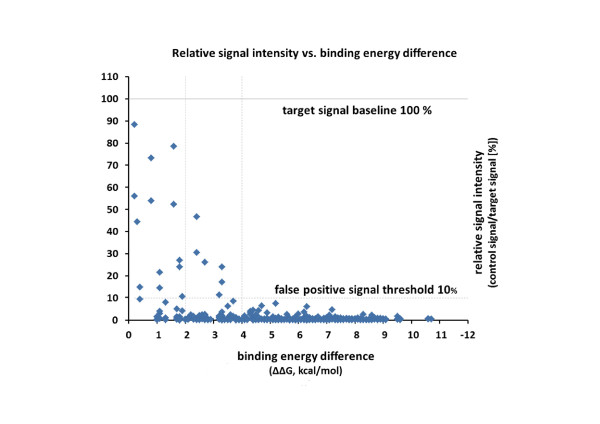
**Non-specific microarray signal intensities from hybridization experiments with target and five control tmRNAs**. Non-specific tmRNA signal intensities are divided by the corresponding probe-specific signal intensity. Target hybridization signal intensity is given on a y-axis as a 100% signal baseline. A maximum false-positive signal threshold is shown as horizontal 10% dotted line. Microarray signals are distributed along x-axis according to the calculated binding energy difference between the specific and non-specific binding (ΔG_target _- ΔG_control _(ΔΔG) 0.2...10.7 kcal/mol). Dotted vertical lines separate the probes with binding energy difference smaller then 2 and 4 kcal/mol, respectively.

**Figure 2:**
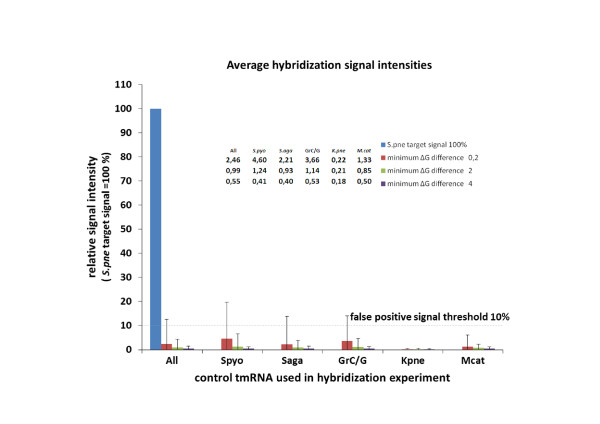
**Average microarray signal intensities with target *Streptococcus pneumoniae *and five different control tmRNAs**. The probe-specific target tmRNA hybridization signal average is shown as a 100% bar. Control signal averages of five different tmRNAs (*Streptococcus pyogenes (S.pyo), Streptococcus agalactiae (S.aga)*, GrC/G streptococcus (GrC/G)*, Klebsiella pneumoniae (K.pne) *and *Moraxella catarrhalis (M.cat)) *are given as a percentage of the target signal. Three different average bars for control tmRNAs represent the average hybridization signal intensities with probes' minimum ΔG differences 0.2; 2 and 4 kcal/mol compared to the hybridization with target molecule (ΔΔG = 0). Error bars show SD of control signal averages. All of the average signal values of the control tmRNA hybridization reactions are shown on the table added onto the graph.

A series of hybridization experiments were conducted to validate the SLICSel program by testing the specificity of the SLICSel designed oligonucleotide probes and their suitability for the use in development of diagnostical technology. In total 97 oligonucleotide probes were designed complementary to the different regions of *S.pneumoniae*'s tmRNA (the main target molecule). Control tmRNA molecules were from five other bacteria: *S.pyogenes, S.agalactiae*, GrC/G streptococcus, *K.pneumoniae *and *M.catarrhalis*. All tmRNA sequences were synthesized i*n vitro *and then hybridized individually to the panel of *S.pneumoniae *tmRNA specific probes on microarray. Figure 1 shows the scatter plot of relative signal intensities of control tmRNA hybridizations onto microarray probes according to their binding energy difference ΔΔG between target and control RNA. From a total of 463 hybridization events only 20 (~4.3%) gave relative signal intensities higher than preset 10% false positive signal threshold condition. For the remaining 443 hybridizations (95.7%) the control signals remained under the threshold level. As shown in the Figure 1, designing probes with higher binding energy difference (ΔΔG) decreases the possibility of a false positive signal. For example, choosing the probes with the minimum ΔG difference of 4 kcal/mol was sufficient to avoid all the false-positive bindings over the threshold while in the case of ΔG difference 2 kcal/mol 6 signals remained over the 10% signal threshold (~1.5% of hybridizations). The average hybridization signal intensities of target and control tmRNAs (all five together and individually) are shown on a bar chart and complementary table in Figure 2. Nearly fivefold increase of the probe specificity was achieved with ΔΔG condition 4 kcal/mol as the average false-positive control tmRNA signal intensity dropped from 2.46% to 0.55%. All of the average false-positive hybridization signals of individual tmRNAs were lower with higher minimum ΔΔG criteria. In general, control tmRNAs from bacteria belonging to the Streptococcus genus showed stronger than/or near average false-positive hybridization signals while signals of more distant *K.pneumoniae *and *M.catarrhalis *remained under the overall average. *K.pneumoniae *tmRNA produced lowest average false-positive signals in all three different minimum ΔΔG conditions and had no signals over the 10% threshold. All of the false-positive signals greater than 10% were contributed by 10 single microarray probes. After removal of those problematic probes the average hybridization signal intensities were under 1% for all the different control tmRNAs.

### NASBA-microarray technology

**Figure 3:**
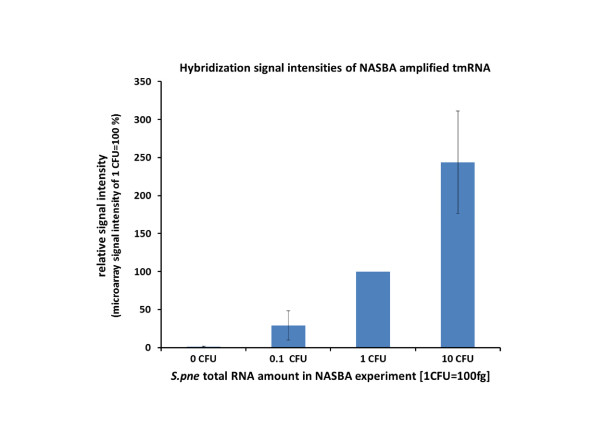
**Microarray signal intensities of NASBA amplified tmRNA from *Streptococcus pneumoniae *total RNA dilutions**. The microarray signal intensity of NASBA amplified 1 CFU total RNA was set as a 100% in all three parallel experiments. Rest of the RNA dilution hybridization signals from 0 CFU (equal volume of NASBA water as negative control), 0.1 and 10 CFU represent their relation to 1 CFU signal as a percentage. Error bars show ± 1 SD of signal averages over three parallel experiments. 1 CFU equivalent of total RNA stands for 100 fg of RNA from *S.pneumoniae*.

To test the SLICel designed probes for their potential use in microbial diagnostics; a new microarray was designed that consisted of the 25 best performing probes out of 97 according to their specificity and the sensitivity in the validation experiments. For control purposes oligonucleotide probes specific to *S.pyogenes, S.agalactiae*, *K.pneumoniae *and *M.catarrhalis *were also added to the microarray. tmRNA molecules of *S.pneumoniae *were amplified from three different total RNA dilutions (equaling to 0.1, 1 and 10 CFU, respectively) and labeled for microarray hybridization. Microarray signals were obtained with all three total RNA dilutions in all of the three parallel experiments including the 10 fg of total RNA sample equivalent to 0.1 CFU. According to the total RNA input into the NASBA reaction, microarray signals increased correspondingly with 0.1 CFU being the lowest and 10 CFU the highest in three replicate experiments (figure 3). Hybridization experiments with NASBA amplified negative control solution provided no significant signals over the background level on microarray. NASBA control experiments with excess amounts of total RNA mix from 4 control species (*S.pyogenes, S.agalactiae*, *K.pneumoniae *and *M.catarrhalis*) were performed to verify the specificity of the NASBA-microarray based detection method. 10 pg of total RNA from each of the control species were added, making the background RNA ratio to target RNA 4 × 10:1, 4 × 100:1 and 4 × 1000:1, respectively. Addition of control total RNA-s to NASBA reaction did not cause any changes to the microarray signal intensities; all of the *S.pneumoniae *target dilutions were amplified and detected on the microarray while the negative control remained blank. The capability of the described NASBA-microarray method to detect tmRNA from low amounts of bacteria was also confirmed experimentally when the total RNA was prepared from dilutions of *S.pneumoniae *cultures (0.1 to10 CFU) instead of using total RNA dilutions, making the experiment setup closer to real-world diagnostic situations where only small amounts of target bacteria may be present.

## Discussion

We selected tmRNA as a marker molecule for technological tool development in bacterial diagnostics because they are present in all bacteria [1,2] in high copy number and they contain both conserved as well as highly divergent regions [3]. Presence of intact RNA molecules can additionally indicate the viability of the bacterial population in the analyte solution [26]. These characteristics make tmRNA a suitable marker molecule in microbial diagnostics. Although the aforementioned properties also apply to16S rRNA (and its corresponding gene), possibly the best known and most used marker in diagnostic and phylogeny studies, the need for investigation of novel alternative marker molecules like tmRNA remains as 16S rRNA often cannot be used to detect and distinguish closely related species [4,31]. For microarray-based detection technologies, the signal strength is determined by the number of target molecules hybridized to probes, i.e. by the equilibrium point of hybridization, and can thus be theoretically predicted using the nearest-neighbor thermodynamic model. The same model, incorporating mismatches, can also be used to predict the signal strength of nonspecific hybridizations - i.e. false-positive signals. In our approach the goal was not to design probes with maximum affinity, but instead maximize the difference of affinity between specific and nonspecific hybridization at annealing temperature. The microarray hybridization experiments conducted with tmRNA specific probes gave information about the concept of designing probes using NN thermodynamic modeling in SLICSel and whether the tested probes are suitable for further species detection and identification. In general the hybridization experiments with *in vitro *synthesized target and control tmRNA molecules proved that SLICSel designed probes are highly capable of specific bacterial identification. By implementing stringent binding energy difference criteria during probe design SLICSel can minimize the possibility of designing probes that would result in false-positive signals. In our validation experiment the hybridization binding energy difference ΔΔG 4 kcal/mol between control and target tmRNA was sufficient to eliminate all the false-positive control signals over the needed threshold level (Figure 1). We achieved an almost fivefold increase in average probe specificity by using stringent ΔΔG criteria 4 kcal/mol (Figure 2). Although, the efficiency of average SLICSel designed probe is high, there is no 100% guaranteed approach for the *in silico *oligonucleotide probe design for hybridization based experiments with surface-immobilized probes. Additional probe specificity evaluation *in vitro *and low quality probe removal still remain as necessary steps in any microarray experiment [20]. In our case the removal of 10 probes was needed to assure that hybridization signals with control tmRNAs remain safely under the determined 10% threshold level. We designed a new microarray incorporating only the optimum *S.pneumoniae *specific probe sequences for the detection of labeled tmRNA products amplified using NASBA. A key characteristic of the NASBA-microarray technology, especially in microbial diagnostics, is that the detection and the identification of the correct target can be optimized at two different points in the experimental protocol. The selection of oligonucleotide primer set determines the specificity of the NASBA amplification phase while a second level of specificity is provided by the SLICSel designed immobilized microarray probes. Specific amplification of a single RNA molecule or wider selection of various RNAs in case of multiplex-NASBA is possible. Certain rules have been described for the NASBA primer pair design [32], but as no convenient software has yet been developed it remains somewhat a trial-and-error approach. In our case the primer set was designed according to the aforementioned rules to amplify a near full length tmRNA molecule from *S.pneumoniae*. We included additional control probes specific to *S.pyogenes, S.agalactiae*, *K.pneumoniae *and *M.catarrhalist *in the microarray to determine the specificity of NASBA amplification step conducted in the presence of a non-*S.pneumoniae *total RNA background. The composition of capture probes on the microarray depends on the overall goal of the experiment. In our case the objective was to specifically detect tmRNA molecules from *S.pneumoniae *total RNA and test the sensitivity of the method previously described by us [29]. Our intention was to investigate whether the method is capable of detecting 1 CFU by using tmRNA as a target molecule. Previous works have shown that detection of 1 CFU by using NASBA amplification of rRNA [33] or tmRNA [6] is possible. The addition of highly parallel microarray based detection to this amplification technology could represent a significant advance in microbial diagnostics; particularly in situations where high number of different bacterial species may be present (such as environmental samples) or in clinical settings where it is necessary to identify one particular infection causing species from a large panel of potential pathogens. We successfully detected and identified *S.pneumoniae *tmRNA molecules from all three different dilutions of total RNA used in experiments (Figure 3). Our experiments proved that 0.1 CFU equivalent total RNA was sufficient to produce strong reproducible hybridization signals on our microarray. Addition of background total RNAs to the NASBA reaction mix provided no signals on control probes on microarray, confirming the high specificity of NASBA-microarray technology and also its components: NASBA primers and microarray probes. In case of the specific tmRNA detection from 0.1 CFU equivalent of *S.pneumoniae *total RNA, the amount of non-specific RNA exceeded the target 4000 times. The described high level of achieved specificity and sensitivity demonstrates the potential and suitability of NASBA-microarray technology for the purpose of pathogen detection in microbial diagnostics or more complex analysis of microbial taxa in environment.

## Conclusions

We have presented a novel technological procedure for bacterial diagnostics and microbial analysis. The nearest-neighbor thermodynamics based SLICSel tool is not exclusive for tmRNA and microarray probe design, but can be used for any other hybridization based technology where DNA or RNA oligonucleotide probe design is necessary. The combination of NASBA amplification technology with microarray based fluorescently labeled RNA detection enabled us to detect tmRNA molecules from as low as 0.1 to 10 CFU of *S.pneumoniae *total RNA. Using the described approach different patient samples, food products or any analyte solution can be tested and screened in a highly parallel approach for several live pathogens or contaminants. SLICSel and NASBA-microarray technology can be used separately for different areas of microbial diagnostics including environmental monitoring, bio threat detection, industrial process monitoring and clinical microbiology.

## Authors' contributions

OS conducted NASBA and microarray experiments, performed microarray analysis and drafted the manuscript. LK designed SLICSel and microarray probes, helped with microarray data analysis and drafted the manuscript. BG carried out the microbiological experiments and RNA extraction and helped to draft and review the manuscript. PP helped designing SLICSel and the microarray probes, helped in data analysis and drafted the manuscript. SP participated in NASBA and microarray experiments and data analysis. KT participated in NASBA and microarray experiments and helped in manuscript review. MM and TB conceived of the study, participated in its design and coordinated microbiological experiments. MR conceived of the study and participated in its design, conducted SLICSel design, helped to draft and review the manuscript. AK conceived of the study, participated in its design, coordinated NASBA and microarray experiments and helped to draft and review the manuscript. All authors read and approved the final manuscript.

## Supplementary Material

Additional file 1 **Methods supplementary file**. Additional file describing thoroughly all of the necessary data and reagents needed for the methods section Click here for file
