# Supplementary material for: Detection of NASBA amplified bacterial tmRNA molecules on SLICSel designed microarray probes
Source: BMC Biotechnol. 2011 Feb 28;11:17. doi: 10.1186/1472-6750-11-17 (PMC3051898; doi:10.1186/1472-6750-11-17)
Supplement: Additional file 1 — Methods supplementary file. Additional file describing thoroughly all of the necessary data and reagents needed for the methods section [file 1472-6750-11-17-S1.DOCX]

**Additional file 1**

**Title: Methods supplementary file**

**Description: Additional file describing thoroughly all of the necessary data and reagents needed for the methods section**

**Bacterial strains and total RNA extraction**

Total RNA was purified from overnight cultures using RiboPure Yeast Kit (Ambion, Austin, TX, USA). Although the RiboPure Yeast kit is not originally intended for RNA extraction from bacteria, high quality bacterial total RNA was obtained without making any modifications to manufacturer's instructions. In order to enumerate the CFUs for NASBA experiment, duplicate 100 μl aliquots of each dilution were plated onto Columbia Chocolate agar (LIP Fanin, Galway, Ireland) and incubated overnight at 37°C in a gas jar under anaerobic conditions. Following overnight incubation, plate counts were performed to determine the cell load of each of the RNA extractions.

**Microarray design**

Probe length varied between 9 and 26 nucleotides (average 16), melting temperature (Tm) between 53°C and 60°C (average 58°C) and binding energies (ΔG) with complementary tmRNA were predicted to be between 17 kcal/mol and 30 kcal/mol (average 23 kcal/mol) at 45°C and in 50 mM salt. To validate the SLICSel design concept, the 97 S.pneumoniae-specific probes had wide scale of ΔG differences between specific and nonspecific hybridization (0.2…10.7 kcal/mol) with control tmRNA sequences. The average ΔΔG with control tmRNAs was 5,4; individually with *S.pyo*, *S.aga*, GrC/G, *K.pne* and *M.cat* 5.2; 5.3; 5.0; 5.7 and 5.9 respectively. All of the ΔΔG values were identical for tmRNA sequences of Group C and Group G streptococcus due to their almost identical similarity and therefore they are referred to as Group C/G in the manuscript (GrC/G). All the probes designed were tested using Mfold <http://mfold.bioinfo.rpi.edu> to exclude those with potential secondary structures, and MegaBlast <http://www.ncbi.nlm.nih.gov/blast/megablast.shtml> to eliminate possible cross-hybridization with unwanted targets, including tmRNAs from other species, bacterial DNA/RNA and human genomic DNA or RNA sequences. Three extra control probes with complementary fluorescent targets (spikes) were designed for normalization. Microarray probes with 5'amino modifications and C6 spacers were diluted in 100 mM Na_2_CO_3_/NaHCO_3_ (pH 9.0) to 50 μM final concentration and spotted onto SAL-1 Ultra microarray slides in Asper Biotech Ltd. (Tartu, Estonia). Each slide contained four datapoints for each probe as two identical sub-grids were spotted with duplicate spots. Probe list available as additional material in previously published article <http://www.biomedcentral.com/1472-6750/10/34/additional/>

Probes selected for NASBA-microarray experiments in current manuscript:

1. 25 *Streptococcus pneumonia* specific probes:

1_11, 2_3, 2_4, 2_5, 2_6, 3_3, 3_11, 3_12, 3_13, 3_14, 3_15, 3_16, 4_1, 4_17, 4_20, 4_22, 4_23, 4_24, 4_25, 4_26, 4_27, 4_28, 5_1, 5_2, 6_3

1. 3 *Streptococcus pyogenes* specific probes: s_p4_1, s_p5_1, s_p5_4
2. 3 *Streptococcus agalactiae* specific probes: s_a1_1, s_a4_3, s_a5_2
3. 3 *Klebsiella pneumoniae* specific probes: k_p2_6, k_p5_11, k_p1_28,
4. 3 *Moraxella catarrhalis* specific probes: m_c1_4, m_c1_6, m_c1_21

***In vitro* tmRNA synthesis for validation experiment**

The *ssrA*-containing vector was linearized in 1× buffer R using either HindIII or XhoI restriction endonuclease depending of the gene orientation in vector (both reagents from Fermentas-ThermoFischer, Vilnius, Lithuania). The reaction was carried out for 60 min at 37°C, followed by 15 min enzyme inhibition at 65°C. tmRNA-s were transcribed *in vitro* using 25 ng linearized vector and 20 U T7 or SP6 RNA polymerase (depending of the gene orientation in vector) according to the manufacturer's recommendations. Briefly, final 1× reaction buffer contained 2 mM ATP, 2 mM CTP, 2 mM GTP and 1 mM UTP; 30 U RiboLock™ ribonuclease inhibitor was added to prevent possible RNA degradation. In order to label RNA molecules after the reaction aminoallyl-UTP (aaUTP) was added in 1 mM final concentration, making the final UTP:aaUTP ratio 1:1. A reaction volume of 25 μl was achieved by adding DEPC-treated water. All the reagents were purchased from Fermentas-ThermoFischer. The transcription reaction continued for 120 min at 37°C. *In vitro* synthesized RNA was purified using a NucleoSpin® RNA CleanUp Kit (Macherey-Nagel GmbH, Düren, Germany) according to the manufacturer's protocol. A final 60 μl of the material eluted was vacuum dried in an RVC 2-25 CD rotational vacuum concentrator (Martin Christ GmbH, Osterode am Harz, Germany).

**Table 1 - *Streptococcus pneumoniae* NASBA primers used in current experiment**

| Primer | Sequence 5’-> 3’ |
| --- | --- |
| Forward +T7 | AATTCTAATACGACTCACTATAGGGAGAAGGTTCGACAGGCATTATGAGGCATA |
| Reverse | CGTCCAAACACCTGCCAACATA |

**Labeling of aa-UTP modified RNA and microarray hybridization**

Cy3 (50 nmole) diluted in 2 μl DMSO (Applichem, Darmstadt, Germany) was added to tmRNA diluted in 7 μl 0.1 M Na_2_CO_3_ (pH 9.0). The mixture of RNA and dye was incubated at room temperature for 60 min and the remaining excess Cy3 label was quenched by adding 3.5 μl 4 M H_2_NOH. After the coupling reaction, 35 μl 100 mM sodium acetate was added to neutralize the solution. The labeled RNA was purified with a NucleoSpin^®^ Kit and vacuum dried in an RVC 2-25 CD concentrator.

**Table 2 Microarray hybridization protocol used in an automated HS-400 hybridization station**

|  |  | **Temp. °C** | **Duration** | **Repetitions** |
| --- | --- | --- | --- | --- |
| **1** | Prewash | 85 | Wash: 60 s; Soak: 30 s | 1 |
| **2** | Probe injection | 55 |  | 1 |
| **3** | Hybridization | 55 | 4h 00min, High agitation | 1 |
| **4** | 1.wash | 23 | Wash: 90 s; Soak: 30 s | 3 |
| **5** | 2.wash | 23 | Wash: 90 s; Soak: 30 s | 3 |
| **6** | 3.wash | 23 | Wash: 90 s; Soak: 30 s | 3 |
| **7** | Slide drying | 23 | 90 s | 1 |

- Prewash - 6X saline sodium citrate (SSC); 0,5% Na-dodecyl sulphate (SDS)
- Hybridization - 6X SSC; 0,5% SDS and 5X Denhardts solution
- 1.wash - 2X SSC; 0,03% SDS
- 2.wash - 1X SSC
- 3.wash - 0,2X SSC
